# A NOVEL APPLICATION OF TRANSCRANIAL PULSE STIMULATION IN REHABILITATION: PAIN MANAGEMENT IN REFRACTORY KNEE OSTEOARTHRITIS – A CASE SERIES

**DOI:** 10.2340/jrm.v57.42403

**Published:** 2025-09-23

**Authors:** Marta IMAMURA, Gilson TANAKA SHINZATO, Leandro HEIDY YOSHIOKA, Sabrina SAEMY TOME UCHIYAMA, Beatriz AKEMI TANAKA, Lucas RAMOS DE PRETTO, Felipe FREGNI, Linamara RIZZO BATTISTELLA

**Affiliations:** 1 Instituto de Medicina Fisica e Reabilitacao, Hospital das Clinicas HCFMUSP, Faculdade de Medicina FMUSP, Universidade de Sao Paulo, Sao Paulo, SP, BR; 2 Centro de Pesquisa Clinica, Instituto de Medicina Fisica e Reabilitacao, Hospital das Clinicas HCFMUSP, Faculdade de Medicina, Universidade de Sao Paulo, Sao Paulo, SP, BR; 3 Departamento de Medicina Legal, Bioetica, Medicina do Trabalho e Medicina Fisica e Reabilitacao, Faculdade de Medicina FMUSP, Universidade de Sao Paulo, Sao Paulo, SP, BR, Brazil; 4 Neuromodulation Center and Center for Clinical Research Learning, Spaulding Rehabilitation Hospital and Massachusetts General Hospital, Harvard Medical School, Boston, MA, USA

**Keywords:** knee osteoarthritis, chronic pain, refractory pain, transcranial pulse stimulation, neuromodulation

## Abstract

**Background:**

Knee osteoarthritis is the most common form of arthritis in adults and a leading cause of years lived with disability. Knee osteoarthritis is a significant burden on health systems worldwide.

**Objective:**

This study evaluated the impact of transcranial pulse stimulation in pain intensity on a case series of 8 patients with refractory pain due to primary knee osteoarthritis.

**Design:**

Prospective before-and-after case series.

**Setting:**

Tertiary rehabilitation outpatient clinic at a university hospital.

**Methods:**

Transcranial pulse stimulation was delivered in 6 sessions per participant on 8 patients, diagnosed with knee osteoarthritis using the American College of Rheumatology and the Kellgren–Lawrence radiographic grading criteria, with a nominal weekly interval but an adaptive schedule that accommodated individual and logistical constraints. Overall adherence to the programme, the effect on pain level on the Visual Analogue Scale and side effects were assessed.

**Results:**

In total, 8 female patients were evaluated for the visual analogue scale score before and after therapy. Their ages ranged from 63 to 77 years, with an average of 69.3 (± 5.3) years. The mean initial (before therapy) Visual Analogue Scale score for the right knee was 6.4 (± 2.5) across the patients, and that score reduced to an average of 1.1 (± 1.6) by the end of the therapy. Similarly, the average for the left knee reduced from 7.2 (± 1.4) to 1.4 (± 1.8). This resulted in an average reduction in pain of 5.3 points for the right knee and of 5.8 points for the left knee. All patients improved their scores. Proper adherence and tolerance to the transcranial pulse stimulation protocol was observed, with no severe side effects.

**Conclusion:**

Transcranial pulse stimulation reduced pain in patients with refractory pain due to primary knee osteoarthritis. It may be considered as an intervention for knee osteoarthritis patients with chronic disabling pain.

## LAY ABSTRACT

Knee osteoarthritis is the most common painful condition and a major cause of disability worldwide. Some people continue to experience severe pain even after trying many different treatments. In this study, we explored a new, non-invasive brain stimulation method called transcranial pulse stimulation to help relieve this pain. Eight women with long-standing knee pain received 6 sessions of transcranial pulse stimulation. We found that all patients experienced a significant reduction in their pain scores. Transcranial pulse stimulation was well tolerated, with no serious side effects reported. Our findings suggest that transcranial pulse stimulation may offer a new option for patients with chronic knee pain who have not found relief with other treatments.

Knee osteoarthritis (KOA) is the most common form of arthritis in adults and a leading cause of years lived with disability. It represents a significant burden on healthcare systems worldwide, affecting approximately 600 million in 2020, with a projected increase of 74.9% by 2050 (1).

Osteoarthritis is among the top reasons for people to seek medical care due to chronic pain. In general, any type of chronic pain exerts an enormous personal and economic burden, affecting more than 30% of people worldwide. Chronic pain should be considered a health condition with challenging treatments, as recovery rate is about 5.4% (2). Current recommendations for management of pain due to KOA fail to provide clinical improvements, consequently increasing the number of knee replacements and revision of these replacements.

The Institute of Physical Medicine and Rehabilitation (IMREA) comprehensive rehabilitation programme provided pain relief and functional improvement in most patients (3). However, some patients failed to improve their pain symptoms over time.

Transcranial pulse stimulation (TPS) is a relatively new, non-invasive brain stimulation technique that uses ultrashort shock wave pulses to target specific cortical areas of the brain. This method has been developed to modulate neural activity, and preliminary studies show that TPS can induce significant therapeutic effects in Alzheimer’s disease (AD) (4–12). The use of this intervention for treating chronic osteoarthritis pain is an innovative approach rooted in extensive research on the effects of shockwaves on brain tissues. Therefore, this pilot case series assessed the effects of TPS to improve pain symptoms in patients with primary KOA refractory to conventional interventions.

## METHODS

This is a prospective pre–post intervention case series study assessing the impact of TPS for community-dwelling adults with KOA. The study was conducted at the Institute of Physical Medicine and Rehabilitation (IMREA) Hospital das Clinicas University of Sao Paulo School of Medicine (HCFMUSP) from December 2023 to May 2024. Participants with a clinical and radiological diagnosis of KOA were invited to participate in the study and included after signing the informed consent form previously approved by the Hospital das Clinicas da Faculdade de Medicina da Universidade de Sao Paulo Ethics Committee for Research, Protocol Analysis CAAE: 86832518.7.0000.0068.

### Participants

Patients referred to IMREA for pain related to KOA were screened to participate in the study. Investigators assessed eligibility for the study by reviewing medical records, radiological studies, and patient interviews. The inclusion criteria included patients with moderate to severe pain related to KOA, with a Visual Analogue Scale (VAS > 4), of at least 3 months’ duration, and who met the American College of Rheumatology and the Kellgren–Lawrence radiographic grading criteria for primary KOA.

The exclusion criteria included patients with untreated severe psychological or psychiatric diseases, diagnosed with fibromyalgia, systemic inflammatory rheumatic diseases (including rheumatoid arthritis, haemochromatosis, psoriatic arthritis), post-traumatic KOA, neoplasia, or intolerance or allergy to lidocaine or local anaesthetics. Also, concomitant use of anticoagulant drugs or platelet aggregation inhibitors, intracerebral bleeding, brain tumours, systemic corticosteroid use, or brain implants were excluded from the study.

### Intervention

Each participant received 6 TPS sessions using the Neurolith^®^ device (Storz Medical, Tägerwilen, Switzerland). The treatment required normal ultrasound gel as coupling media over the scalp and hair. The shockwaves were focused on a depth of 5 cm, with maximum energy between 4 and 6 cm. The energy flux density for transcranial application was 0.25 mJ/mm², with a frequency of 4 Hz, delivering a total of 6,000 pulses within 26 min using a slow sweeping motion. Each patient received 1,400 pulses to the precuneus region (700 pulses per hemisphere). The precuneus was localized using the Pz from the international 10–20 electroencephalogram system as reference. The stimulation area was defined around Pz with a diameter of 5 cm latero-laterally and a length of 6 cm antero-posteriorly. Additionally, 800 pulses per hemisphere were delivered over the primary motor cortex (M1), localized from the sagittal midline (Cz) toward the ear, targeting electrodes T3 (left hemisphere) and T4 (right hemisphere), as guided by a neuronavigation system. The remaining 3,000 pulses were distributed bilaterally across broader cortical regions, including the frontal, temporal, parietal, and occipital areas, ensuring that all regions were adequately covered based on the neuronavigation system.

This strategy for chronic pain was based on previous studies with TPS and other forms of neuromodulation, such as transcranial magnetic stimulation (TMS) targeting the M1 cortical area (13) and the pre-frontal cortical area (14).

Precuneus atrophy was documented by brain MRI volumetry in chronic pain patients with KOA (15). Reversal of precuneus atrophy with TPS treatment has also been observed in AD patients (16). These findings led to the hypothesis that stimulating the precuneus could alleviate chronic pain by potentially reversing or mitigating this atrophy and enhancing the crucial connections within this area.

Within the diffusely targeted areas were frontal and prefrontal areas, particularly the dorsolateral prefrontal cortex (right and left), which are associated with mood stability and cognitive functions (17). Stimulating these regions may help manage mood-related symptoms often linked to chronic pain conditions. Additionally, the broader brain stimulation was based on the connectome theory, which postulates that all brain regions are interconnected.

The technique is straightforward and easy to apply, with the navigation system providing visual feedback to avoid overstimulation of brain areas, which was indicated by a purple colour on the screen. When this occurred, the operator could quickly adjust the probe to focus on a different area.

### Study measures

Demographic data included age, sex, and presence of systemic arterial hypertension (SAH) and diabetes mellitus (DM).

Pain was assessed by the VAS before and after the programme. A 10-cm VAS was used to measure the pain intensity, and the participants were instructed to mark on the scale where their perceived pain intensity was, such that 0 referred to no pain and 10 the worst pain possible.

Programme adherence was measured by recording the subject’s participation. Side effects were assessed after each session.

### Statistical analysis

Continuous variables are reported as means, with standard deviation, minimum, maximum, and medians tabulated. Categorical variables are reported as percentages followed by the count.

Simple relationship was evaluated by simple and multivariate linear regression models. Correlation was evaluated through Pearson’s method. Finally, significant changes between pairs of measurements were evaluated using a sign test on the difference between those pairs. This test was chosen due to the small number of samples and highly skewed distribution of the differences. We tested the difference from a median of 0. All analyses were conducted in Python (https://www.python.org/) using freely available libraries.

## RESULTS

All patients underwent six sessions of therapy, and the VAS score was measured before the first and after the last therapy session.The participants’ baseline characteristics and duration of their TPS programme are summarized in Table I.

**Table I:** Individual and summary demographic and clinical characteristics (*n* = 8)

Patient	Age (years)	Sex	Diabetes (Y/N)	Hypertension (Y/N)	TPS sessions completed	Interval first ↔ last session (weeks)	KL grade – right knee	KL grade – left knee	Pain duration (years)
1	66	F	N	Y	6	6	III	IV	15
2	73	F	N	Y	6	6	II	II	6
3	63	F	Y	Y	6	6	III	I	2
4	77	F	N	Y	6	40	II	I	7
5	65	F	N	N	6	6	II	III	11
6	75	F	Y	Y	6	6	I	III	21
7	70	F	Y	Y	6	36	III	II	30
8	65	F	N	N	6	11	II	I	20
Mean ± SD/*n* (%)	69.3 ± 5.3	Female 8 (100%)	3 (37.5 %) Y	5 (62.5%) Y	6 ± 0	14.6 ± 13.6	–	–	14 ± 9.3
Median	68.0	–	–	–	6.0	6.0	13		

Numeric data shown per participant; last row gives the mean ± SD (or *n* (%) for categorical variables).

Y: yes; N: no; TPS: transcranial pulse stimulation; KL: Kellgren–Lawrence radiographic grade; F: female; %: percentage; *n*: number of participants.

There was a deviation from the TPS protocol for three patients who could not receive TPS during the six consecutive weeks. Reasons included equipment maintenance, and personal issues with family member and holiday seasons, requiring longer intervals among sessions. Consequently, the total treatment course spanned 6–40 weeks (median 6).

All patients improved their scores. Those results are summarized in Table II.

**Table II:** Individual VAS scores and summary statistics

Patient	Initial R	Final R	Δ R	Initial L	Final L	Δ L
1	4.6	0.0	4.6	7.8	0.4	7.4
2	6.4	4.5	1.9	4.9	3.5	1.4
3	7.7	0.0	7.7	6.8	0.0	6.8
4	8.0	1.3	6.7	8.0	1.1	6.9
5	6.6	2.3	4.3	9.2	4.9	4.3
6	1.1	0.2	0.9	5.6	0.3	5.3
7	9.0	0.5	8.5	7.0	0.4	6.6
8	7.6	0.2	7.4	7.9	0.1	7.8
Mean	6.4	1.1	5.3	7.2	1.4	5.8
SD	2.5	1.6	2.8	1.4	1.8	2.1
Median	7.1	0.4	5.7	7.4	0.4	6.7
Min	1.1	0.0	0.9	4.9	0.0	1.4
Max	9.0	4.5	8.5	9.2	4.9	7.8

R: right knee; L: left knee; SD: standard deviation; Min: minimum values; Max: maximum values; Δ: delta, difference between initial and final values.

Simple and multiple linear regression models found no significant relationship between age and improvement in the scores for either knee side, even when controlled for the initial VAS score, total interval of sessions, pain duration, or the presence of comorbidities (or any combination thereof). Pearson’s correlation analysis reveals no significant correlation between age and right knee improvement (0.41), and age and left knee improvement (0.31). Nevertheless, given our limited dataset, it may be the case that we are under-powered in those analyses and therefore would not be able to detect significant associations. However, a sign test reports that the median of the improvements for the right (*p* = 0.008) and left (*p* = 0.008) knees are statistically different from 0, which indicates a significant improvement for both knees.

This treatment was painless, and apart from a transient mild headache reported by a single patient during 1 session, no other significant side effects were observed.

## DISCUSSION

In this study, the application of TPS demonstrated significant pain reduction in patients with refractory KOA, supporting its potential as a viable therapeutic option. This aligns with prior research conducted at our institution, where various interventions have highlighted the complex interplay between pain, functional impairment, and neuroplasticity in KOA. Ultrasonography findings have underscored the role of periarticular structures in pain generation (18), while electroencephalogram (EEG) and neuroimaging studies have provided insights into the neurophysiological correlates of chronic pain (19–24), and genetic polymorphism also has an important role in pain expression and response to treatment (25).Together, these studies suggest that a multimodal approach, incorporating both peripheral and central mechanisms, may be essential for effectively managing chronic KOA pain.

Shockwaves act at cellular level by mechanotransduction, biochemical responses to mechanical stimuli of transmembrane proteins called integrins (26–28), and studies highlight this effect on the development and function of neural cells, with special mention of hippocampal neurons and astrocytes (29–31).

TPS was conceived and first described in the recovery of severe brain injuries, with unresponsive wakefulness syndrome (32).

Various physiological effects on brain tissue have been demonstrated with this intervention, including the release of neurotrophic factors such as brain-derived neurotrophic factor (BDNF), glial cell-derived neurotrophic factor (GDNF), and vascular endothelial growth factor (VEGF) (33–35). These factors are critical for supporting neuronal survival, growth, and differentiation, as well as promoting angiogenesis and improved cerebral blood flow.

Furthermore, the technique may enhance the function of the glymphatic system (36), improving the cerebrospinal fluid (CSF) flow within glial cells and peri-meningeal lymphatics. The glymphatic system is responsible for clearing metabolic waste products from the brain (37, 38) This improved clearance can mitigate neurotoxic accumulation, thereby supporting overall brain health.

TPS also reduces levels of pro-inflammatory cytokines while increasing anti-inflammatory cytokines, contributing to a shift towards an anti-inflammatory state (39). This shift is particularly important in reducing neuroinflammation, which is often associated with chronic pain and neurodegenerative conditions.

A decrease in electroencephalogram (EEG) global Tsallis entropy following the treatment has been documented (40), suggesting a reset of uncoordinated and pathological neural activity, and indicating a neuromodulation effect caused by a mechanical stimulus, diverging from classical electrical or magnetic stimulation (41). Modulation of somatosensory evoked potentials was also documented (5). Additionally, functional magnetic resonance imaging (fMRI) studies have demonstrated enhanced neuronal connectivity and communication, leading to improved structural integrity of brain regions involved in pain processing and modulation (42–44). This could result in prolonged pain relief and functional improvements for KOA patients.

Neuropsychiatric and cognitive improvements with TPS have been documented in studies on AD (4–8, 10–12) and mild cognitive impairment (9).

Clinical improvement was observed in Parkinson’s disease (45, 46), depression (8, 10), autism (47), and attention-deficit/hyperactivity disorder (48).

### Safety issues

Safety studies, with physical and computational measurements of energy levels within the brain tissue, were developed (49, 50). Also, experimental models documented high safety margins for TPS (150-fold) regarding human maximum energy flux density allowed in clinical use (5).

A systematic review on the safety of neuromodulation interventions in the paediatric population with mental disorders or neuropsychiatric disorders included 56 studies, 2 of them of TPS, and found the most common adverse events were headache and general pain or discomfort (51). The TPS Therapy and Development Center in Vienna, Austria made an analysis of a consecutive case series with over 80% of patients reporting no sensation of pressure or pain and no major adverse events (52).

A survey in 35 centres for AD treatment with TPS analysed 3,318 patients and 16,352 treatment sessions, and no side effects were observed in 85% of the centres. Those observed, such as headaches, dizziness, and fatigue, were typically mild, lasting a few hours (53).

This body of evidence, with promising results in various neurological conditions and initial reviews (11, 12), provided a foundation for its potential benefits in chronic KOA pain. Enhanced neuronal connectivity and communication, leading to improved structural integrity in brain regions involved in pain processing and modulation, and the reduction of brain atrophy, particularly in the precuneus (16), could result in prolonged pain relief and functional improvements for patients with KOA, encouraging us to apply TPS in refractory KOA patients, who had an average pain duration of 14 years.

The limitations of this study include the absence of a control group, a small sample size, and the lack of additional scales to evaluate the outcomes. Additionally, there were no long-term follow-ups, making it difficult to assess the durability of the treatment effects. Future studies should incorporate neuroimaging assessments and include larger, controlled trials to better understand the long-term efficacy of this intervention in treating KOA.

### Conclusion

TPS provided significant pain relief after just a few treatment sessions. The degree of pain reduction in such a short period was a striking and unexpected finding in this initial case series.

This intervention showed promise in refractory cases of pain due to primary KOA, especially in patients who failed to respond to the comprehensive rehabilitation programme at IMREA. The significant reduction in pain observed after the treatment enabled patients to initiate exercise and physical fitness activities that they had previously been unable to perform.

TPS effectively reduced pain in patients with refractory primary KOA and may be considered a viable intervention for KOA patients experiencing chronic, disabling pain.
